# Monthly Summary

**Published:** 1878-05

**Authors:** 


### MONTHLY SUMMARY.

*Carbonic Acid Gas Baths*, are used at *Vichy*, for gout, rheumatism, neuralgia, &c., and for uterine troubles, with success. The administration by the stomach does not seem to be successful.

*Relation to Life of Aerostatic Pressure.*—The notion that the fatal effects experienced from exposure to a highly rarefied atmosphere is caused by the diminished pressure, is disputed by Prof. Paul Bert, of Paris, in an essay which has been awarded the great prize, issued every two years by the Institute of France. By an ingenious series of experiments, Prof. Bert shows that when a bird, placed under a bell-glass from which the air is gradually exhausted, it may be immediately restored by the addition of a little pure oxygen to the highly rarefied medium; while by continuing the action of the air-pump, as well as the admission of oxygen, the rarefaction may be kept at a standard supposed to be sufficient to cause death, without resulting in serious inconvenience to the animal. After having experimented upon various animals, Prof. Bert placed himself within a large cylinder, with the nozzle of an oxygen bag in his hand, and caused the air to be slowly exhausted. At the expiration of twenty one minutes, with a barometrical pressure of 590 millimetres, his pulse was 70. At the expiration of thirty-five minutes, with a pressure equivalent to that of 13,000 feet above the sea-level, his pulse was 84 and a feeling of nausea nearly overcame him. Two minutes later the nausea disappeared, the abdomen became a little swollen, and signs of congestion appeared in the face. After being in the cylinder two hours and forty minutes, the pressure being 430 millimetres, and the pulse 84, he took three whiffs of oxygen, and the pulse instantly descended to 78. Four minutes later, he inhaled more oxygen, the disagreeable symptoms vanished, and the pulse went down to 70. In another curious series of experiments, the professor finds that aerostatic pressure, even of oxygen itself, extinguishes life, and is destructive to organic compounds. Ferment organisms, such as bacteria, are almost instantly killed by the action of compressed air, or of compressed oxygen gas, while fermentations due to dissolved matter, such as diastose, perfectly resist the influence of atmospheric pressure. The ripening of fruit being arrested by compressed oxygen, Prof. Bert concludes that the phenomenon is one of cellular evolution. The poison of the scorpion, on the contrary, wholly resisting the action of increased aerostatic pressure, he concludes to be due to the pressure of compound similar to the vegetable alkaloids. Fresh vaccine lymph, after having been subjected to the pressure of 50 atmospheres for a week, retained its virtue; and Prof. Bert concludes from this fact, that its active principle is not the cell found in it, as many have supposed. The poison of glanders remained active after similar treatment, and carbunculus blood, though freed from bacteria by aerostatic pressure, still retained its dangerous properties. He concludes, therefore, that the bacteria, germs, cells, penicilium spores, etc., found in these poisons, are not the cause of their virulence.—*Med. Record.*

*Development of Tænia Solium in Man.*—M. Redon is a brave man. He wished to settle the question as to whether the cysticercus of man was identical with the cysticercus of the pig, and so he swallowed four cysts taken from the body of a dead man. He also administered some of the cysts to sucking pigs and dogs. The pigs died from enteritis and disclosed no worms, the dogs failed to develop the parasites. But M. Redon had himself the satisfaction of finding links of the worms in his passages at the end of three months and two days. These experiments settle the question that has been in dispute so long, that the tænia of man is the second stage of that of the pig in the negative, or at least show that in this case what was ordinary supposed to be the perfect tape worm could reproduce in another man. The law has seemed to be that the same parasite could not attain its complete development in the same individual or in two individuals of the same species.

---

*Dr. Pierce's Golden Medical Discovery*, according to a German chemist, consists of 15 parts clarified honey, 1 part extract of lettuce, 2 parts laudanum, 100 parts alcohol, and 105 parts water. This is to cure long standing coughs, inflammations, scrofulous and syphilitic diseases.

---

*Cerebral Localization*,—Dr. Brodhead, (International Medical Congress of Geneva,) gives the following conclusions as to brain function and lesion:

1. Paralysis is a rupture of fibres or cellules presiding over the mechanism of the nervo-motor apparatus.
2. Anæsthesia is rupture of sensitive mechanism.
3. Tremor is some impediment in the conducting power of the white fibres.
4. Convulsions, (including chorea) result from irritation of gray substance.
5. Premature and transcient contraction is connected with pressure on a ganglion.

Prof. Shiff said he did not believe in motor points in the brain, and that the *role* assigned to the corpora strata and optic thalami had not been proven by clinical investigation.

---

*The Safety Pocket.*—A lively personal discussion arose in the New York Odontological Society, from a paper read by Dr. J. A. Clowes, entitled "The Safety Pocket," which purported to treat with the subject of devitalizing dental pulps by means of arsenic secured in a "safety pocket," drilled in proximity to the nerve. A greater portion of the paper was taken up, however, by a tirade against all modern dental practice, characterizing

"the *remorseless rubber*, the *swelling wood*, the *driven wedge*, the *jack-screw*," as "offshoots of ignorance and relics of barbarism." The application of an arsenical paste directly to the diseased nerve, owing to its liability of coming in contact with the gums, seemed to be particularly displeasing to the Dr., who gravely avowed that "the victims to arsenical misuse and abuse are all around us; where civilization extends they abide, and gum and osseous tissue—fistulated and cavernous, necrosed and sloughing—are reeking with purulence and fetor!" Of course, these expressions could not be allowed to remain unnoticed, and several gentlemen took occasion to freely and severely criticize the paper, Dr. Atkinson remarking that "it is a lack of knowledge of pathology and physiology that makes Dr. Clowes say dentists are doing more harm than good."—*Dental Advertiser.*

---

*Lead Poisoning from a Curious Source.*—The *Medical and Surgical Journal* of New Orleans, has an account of an outbreak of lead poisoning in that city, the source of which was for a long time hidden in mystery. Sixty-five cases were observed, whole families being sometimes affected. Dr. Ducamp, to whom much credit is due for his patient and thorough exploration of the cause, having ascertained that the water supply was good, and that the "wine so universally used" was free from lead, discovered at length that the persons injured had all got their supply of bread from a certain bakery. "It is a well known fact," says the narrative, "that many bakers, to whiten their bread, add the sub-acetate of lead to the dough made with damaged flour." But the recognized honesty of the baker in this instance precluded all suspicion of such a trick. Dr. Ducamp finally observed that the baker heated his oven with wood from buildings which had been demolished in opening a new street. The wood was heavily painted and the lead of the paint, when the wood was burned, effected a lodgment on the floor of the oven. in the form of oxide. When the loaves were placed on the floor of the oven, the poisonous oxide adhered to them, and the lead was distributed in this manner over the city.—*Pacific Medical Journal.*

---

*Relation of Brain Weight to Mental Ability.*—Mr. C. Clapham says, in the last volume of the West Riding Lunatic Asylum Reports:—

"My observations agree with those of Wagner, that weight of brain does not indicate any close relation to intellectual power, and also that aboriginal races are not to be distinguished for smallness of brains. In fact, the ancient Britons, and I may add the ancient Gauls also, were remarkable for good-sized, nay, even lagre brains." This statement is borne out by the testimony of the most competent craniologists of the day.—*Med. and Surg. Reporter.*

